# Unlocking nitrogen regulation: structural insights into the NifL‐NifA complex and prospects for engineered diazotrophs

**DOI:** 10.1111/febs.70332

**Published:** 2025-11-16

**Authors:** Edileusa Cristina Marques Gerhardt, Khaled A. Selim

**Affiliations:** ^1^ Department of Biochemistry and Molecular Biology Universidade Federal do Paraná Curitiba Brazil; ^2^ Microbial Biochemistry Group, Institute of Phototrophic Microbiology, Heinrich Heine University Düsseldorf, Universitätsstr. 1 Germany

**Keywords:** *Azotobacter vinelandii*, biofertilizers, biological nitrogen fixation, NifL‐NifA system, structural biology, sustainable agriculture

## Abstract

The urgent need for sustainable agriculture places biological nitrogen fixation at the forefront of current biotechnological research. Plant growth‐promoting rhizobacteria play crucial roles in agriculture by enhancing nutrient absorption, regulating hormonal balance, and providing reduced nitrogen to plants. Among these, diazotrophic bacteria, such as *Azotobacter vinelandii*, stand out for their ability to fix atmospheric nitrogen and release it in bioavailable forms. In this issue of *The FEBS Journal*, scientists mapped the interaction between NifL and NifA proteins, which regulate nitrogen fixation in *A*. *vinelandii* and many other Proteobacteria. This understanding will allow for engineering bacteria to enhance nitrogen delivery to plants by improving nitrogen fixation.

## Graphical abstract

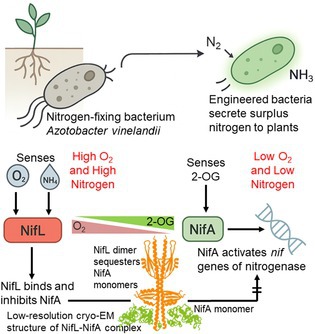

**Abbreviations**
2‐OG: 2‐Oxoglutarate
*A. vinelandii*
: *Azotobacter vinelandii*
AAA+: ATPases associated with various cellular Activities domain
ATP: adenosine triphosphate
BNF: biological nitrogen fixation
cryo‐EM: cryo‐electron microscopy
DNA: deoxyribonucleic acid
GAF: GMP‐Adenylyl cyclase‐FhlA
GHKL/DHp: Gyrase, Hsp90, Histidine Kinase, MutL/Dimerization and Histidine phosphotransfer domains
N_2_
: dinitrogen
NH_3_
: ammonia
PAS: Per‐ARNT‐Sim domain
PGPR: plant growth‐promoting rhizobacteria
TCA: tricarboxylic acid cycle
*σ* ^54^
: Sigma factor 54

## The nitrogen challenge in agriculture

Nitrogen is essential for all living organisms as a fundamental component of proteins and nucleic acids. Despite the abundance of dinitrogen (N_2_) in the atmosphere, its biological inaccessibility has historically limited agricultural productivity. The Haber–Bosch process for ammonia (NH_3_) production from N_2_ and hydrogen revolutionized food production, but at the cost of high‐energy consumption and environmental damage, making up about 1.5% of worldwide greenhouse gas emissions [1]. The search for alternatives has become essential, particularly biofertilizers that are based on nitrogen‐fixing bacteria [2].

**Fig. 1:**
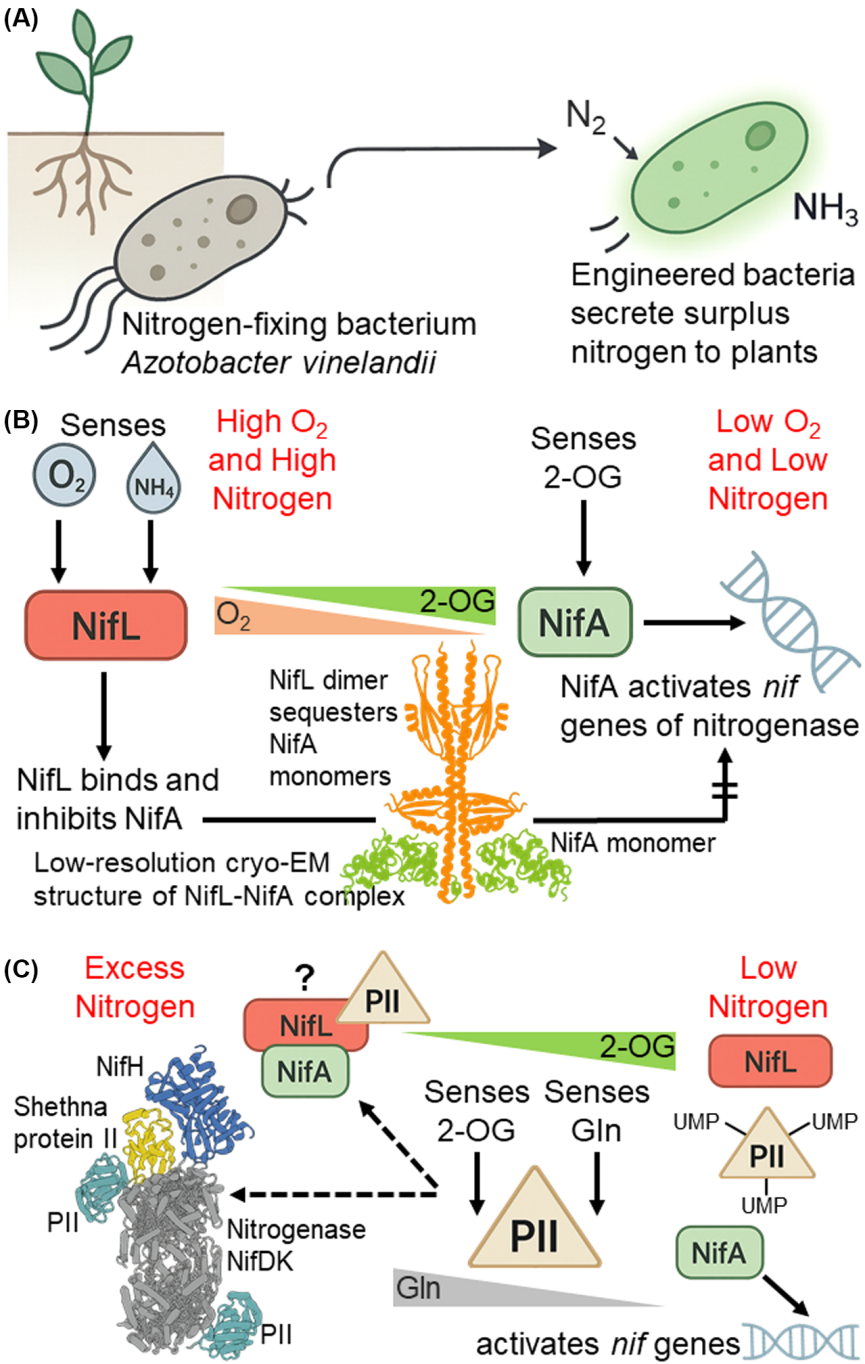
Model for regulation of nitrogenase by NifA, NifL, Shetha protein II and PII proteins, in response to cellular levels of O_2_, 2‐oxoglutarate (2‐OG) and glutamine (Gln). (A) Conceptual representation of engineering a super strain of *A. vinelandii* designed to excrete excess fixed nitrogen into the plant rhizosphere to enhance nitrogen availability. (B) Mechanism of NifL‐NifA regulation in response to oxygen. Under high O_2_ conditions, NifL interacts with NifA and sequesters its monomers, preventing the assembly of the active hexameric NifA and thereby blocking *nif* gene transcription. Under low O_2_ and nitrogen‐limiting conditions (indicated by high 2‐OG), NifA forms an active hexamer that binds promoter regions of *nif* genes to activate nitrogenase transcription. (C) Integration of PII signaling and additional regulatory proteins into the model of nitrogenase regulation. PII protein senses intracellular nitrogen and carbon status via Gln and 2‐OG levels. Under nitrogen excess (indicated by high Gln and low 2‐OG), PII either forms a complex with NifL‐NifA to reinforce inhibition of NifA, or its homolog NifI binds to the nitrogenase complex (NifDK), preventing the association of NifH to keep the nitrogenase inactive. In some cases, the recently described Shethna Protein II protects the oxygen‐sensitive nitrogenase from O2 excess. Under nitrogen limitation (indicated by high 2‐OG and low Gln), PII becomes uridylylated (PII‐UMP) and dissociates from the inhibitory PII‐NifL‐NifA complex. At the same time, NifA senses 2‐OG and increases its affinity for DNA, promoting *nif* gene expression and nitrogenase activity.

Free‐living diazotrophs represent a viable biological strategy for achieving sustainable nitrogen supply into agroecosystems. However, nitrogenase, the key enzyme for biological nitrogen fixation (BNF), is an oxygen‐sensitive and energetically demanding enzyme. Moreover, its cellular production and activity are tightly regulated. Transcriptionally, the *σ* ^54^‐dependent enhancer‐binding protein NifA activates the *nif* gene cluster encoding the nitrogenase machinery, making NifA the central regulator that integrates cellular oxygen, carbon, and nitrogen signals [3]. Thus, unlocking the regulatory mechanisms that modulate the nitrogenase expression is crucial for developing genetically modified bacterial strains capable of releasing an excess of fixed nitrogen around the plant rhizosphere (Fig. 1A).

## The pivotal role of NifA in nitrogen fixation

The NifA modular structure, which is comprised of an N‐terminal GAF domain that senses carbon status via binding to 2‐oxoglutarate (2‐OG), a central AAA^+^ ATPase motor that drives *σ* ^54^‐dependent transcription, and a C‐terminal helix‐turn‐helix DNA‐binding domain, defines it as the master transcriptional regulator of nitrogenase [3, 4]. Despite its essential role, NifA activity is subject to strict regulatory control; however, the regulation of NifA varies significantly. In some diazotrophs, NifA is controlled by the antiactivator NifL (Fig. 1B), often in conjunction with the PII protein (Fig. 1C), which modulates the NifL‐NifA interaction in response to intracellular nitrogen concentration, reflected by glutamine (Gln) levels [5]. In other species, NifL is absent, and NifA is directly regulated by PII proteins [6]. In both situations, PII proteins act as molecular sensors of nitrogen and carbon availability by binding the key metabolite 2‐oxoglutarate [7, 8], as does NifA (Fig. 1B,C). 2‐Oxoglutarate is a central metabolite of the tricarboxylic acid cycle (TCA), thereby serving as an indicator of carbon and nitrogen status of the cell, reinforcing the universality of NifA as the pivotal core control [9]. On the other hand, NifL integrates signals related to oxygen and nitrogen status and cellular energy levels [10, 11]. In diazotrophs that harbor NifL, under unfavorable conditions such as high oxygen, NifL interacts with NifA to inhibit NifA‐dependent nitrogenase expression (Fig. 1B), thus protecting the cell from unnecessary energy expenditure.

## Structural insights into NifL‐NifA interactions

Until recently, the molecular mechanism by which NifL restrains NifA was unclear. Earlier biochemical studies established that NifL and NifA interact stoichiometrically, with inhibition dependent on nucleotide status [12]. Domain‐level analyses revealed that NifL contains PAS domains that act as redox sensors and C‐terminal GHKL/DHp domains that resemble histidine kinases, but lack phosphotransferase activity [10].

In a new study in this issue of *The FEBS Journal*, Batista and colleagues [1] combined cryo‐electron microscopy (cryo‐EM), structural modeling, and mutagenesis to generate the first structural view of the NifL‐NifA complex. They demonstrated a 2 : 2 stoichiometry in which the NifL dimer sandwiches two molecules of the NifA monomer, blocking its oligomerization into the active hexameric state. The interaction interface of the NifL‐NifA complex involves direct contact between the AAA^+^ ATPase domain of NifA and the GHKL/DHp domains of NifL. This configuration prevents ATP hydrolysis and promoter activation. Although this study provides a valuable first glance into how NifL captures and inactivates NifA, its primary limitations are the low resolution of the cryo‐EM map and the fact that it captures just an asymmetric NifL‐NifA complex, suggesting a highly dynamic and complex regulatory system. Nevertheless, they overcome these limitations elegantly by combining biochemical and molecular biology analyses to support their structural observations.

Interestingly, mutations at the interaction interface of the NifL‐NifA complex disrupted the inhibition of NifA by NifL. Introducing these mutations into *A. vinelandii* caused a constitutive expression of *nif* nitrogenase genes, and therefore, these mutants excrete a remarkable amount of ammonium into the medium. This finding confirmed the mechanistic model and highlighted its biotechnological significance.

## From mechanistic detail to agricultural innovation

The implications of these structural and biochemical insights extend far beyond basic biology. As shown by Mus and colleagues [13], specific mutations of *nifL* can generate ammonium‐excreting strains that deliver bioavailable nitrogen directly to crops such as rice, significantly boosting plant growth. Similarly, alternative genetic approaches such as deletions in ammonium transporters or urease genes also yield strains with enhanced nitrogen excretion [2]. However, deleting regulatory genes often imposes metabolic cost or growth defects. The work of Batista *et al*. [1] provided a more precise framework: Targeted mutations at the NifL‐NifA interface can precisely fine‐tune regulation without fully suppressing it, potentially producing robust strains that fix and excrete nitrogen efficiently. Such rational engineering avoids the undesirable effects of global dysregulation, paving the way for designing diazotrophs optimized for agricultural inoculants.

These findings resonate with recent advances in the structural biology of NifL. Boyer *et al*. [12] employed scattering and mass spectrometry to describe the conformational dynamics of NifL under oxidized and reduced states, further clarifying how environmental signals propagate through its modular domains. Taken together, these studies converge on a detailed molecular picture of the NifL‐NifA switch, knowledge that directly informs synthetic biology strategies for biofertilizer development.

## Broader perspectives and remaining questions

The structural characterization of the NifL‐NifA complex represents a significant advancement in our understanding of nitrogen fixation regulation. However, key questions remain. How do the flexible N‐ and C‐terminal domains of NifA, which are unresolved in the presented cryo‐EM structure, contribute to regulation? How representative are these structural features conserved across diverse diazotrophs? How does the global nitrogen sensor PII integrate into the complex?

Notably, the revolutionary advances in cryo‐EM have also recently highlighted how PII can bind and inhibit the nitrogenase [14], opening new avenues for biotechnological applications to improve BNF. In the same regard, scientists recently discovered that a small oxygen‐sensing protein, Shethna protein II, acts as a bodyguard for the oxygen‐sensitive nitrogenase enzyme [15]. It protects the nitrogenase by forming an oxygen‐shield filament around it, preventing its deactivation from oxygen exposure by rapidly neutralizing the harmful oxidative stress. This detailed structural insight would not have been possible without cryo‐EM.

Addressing these questions will be essential to generalize engineering strategies across different microbial species to improve and design customized biofertilizers for different crop systems. The evolutionary relationship of NifL with sensor histidine kinases [10] also suggests that similar regulatory scaffolds could be co‐opted for other biotechnological applications.

## Concluding remarks

By unveiling the structural principles of the NifL‐NifA interaction, Batista *et al*. [1] provide a foundational advance in nitrogen fixation biology. Their work shows how a detailed mechanistic understanding can be directly translated into practical strategies for sustainable agriculture. As global nitrogen demand increases, such insights may be essential in reducing the dependency on energy‐intensive synthetic fertilizers.

Progress in this area will depend on integrating structural biology, microbial genetics, and plant biology to design microbial inoculants that deliver fixed nitrogen efficiently and predictably. In this effort, the NifL‐NifA system stands not only as a paradigm of signal integration in bacteria but also as a gateway to the next generation of biofertilizers.

## Conflict of interest

The authors declare no conflict of interest.

## Author contributions

ECMK and KAS contributed to the conception, drafting, and critical revision of the commentary.
